# Asymmetry in Time Evolution of Magnetization in Magnetic Nanostructures

**DOI:** 10.1038/srep12301

**Published:** 2015-07-22

**Authors:** Jaroslav Tóbik, Vladimir Cambel, Goran Karapetrov

**Affiliations:** 1 Institute of Electrical Engineering, Slovak Academy of Sciences, Dúbravská cesta 9, SK-841 04 Bratislava, Slovakia; 2 Department of Physics, Drexel University, 3141 Chestnut Street, Philadelphia, Pennsylvania 19104, USA

## Abstract

Strong interest in nanomagnetism stems from the promise of high storage densities of information through control of ever smaller and smaller ensembles of spins. There is a broad consensus that the Landau-Lifshitz-Gilbert equation reliably describes the magnetization dynamics on classical phenomenological level. On the other hand, it is not so evident that the magnetization dynamics governed by this equation contains built-in asymmetry in the case of broad topology sets of symmetric total energy functional surfaces. The magnetization dynamics in such cases shows preference for one particular state from many energetically equivalent available minima. We demonstrate this behavior on a simple one-spin model which can be treated analytically. Depending on the ferromagnet geometry and material parameters, this asymmetric behavior can be robust enough to survive even at high temperatures opening simplified venues for controlling magnetic states of nanodevices in practical applications. Using micromagnetic simulations we demonstrate the asymmetry in magnetization dynamics in a real system with reduced symmetry such as Pacman-like nanodot. Exploiting the built-in asymmetry in the dynamics could lead to practical methods of preparing desired spin configurations on nanoscale.

The symmetry is fascinating property of the nature. Modern theoretical concepts in physics are based on investigation of natural laws guided by known symmetries of the system. The symmetry in magnetism appears very naturally due to dipolar nature of the magnetic field sources. In the absence of external magnetic fields the total energy of the magnetic macroscopic body must be the same if the direction of all constituting dipoles is reversed. This is a consequence of the invariance of total energy functional with respect to inversion of the magnetic dipoles.

Recently, however, there have been experimental observations that contradict the above symmetry-based concept of magnetization reversal. Lok *et al.*^1^ have reported asymmetry in magnetization hysteresis curves of the nickel nanodots at low temperatures. The asymmetry was observed in magnetization hysteresis even at external fields as high as ±10 T. There was a clear lack of the inversion symmetry for the hysteresis loops taken at temperatures below 19.8 K. This asymmetric behavior was lost when the temperature was raised above 20 K. At elevated temperatures the magnetization curves became symmetric, even for magnetic field excursions of down to only ±0.1 T.

Another example of the asymmetric behavior of the magnetization is the experimental demonstration of magnetization reversal in ferrimagnets by a single laser pulse^2^. The asymmetric behavior was attributed to synergistic effect of the two magnetic sub-lattices having distinct dynamical time-scales.

From the theoretical point of view the asymmetric behavior of magnetization was observed in simulations of the magnetic vortex nucleation in nanodots with broken inversion symmetry^3^. In this work the vortex nucleation in presence of external magnetic field was studied. The polarity of the nucleated vortex in these simulations did not change sign when the external field was reversed. Later it was shown that this effect is robust even in the presence of weak external fields opposing the preferred magnetization direction and it was attributed to the asymmetry in magnetization dynamics^4^.

Recently, there was a theoretical proposal of a binary switch based on a symmetric double-well potential landscape that can be controlled by means of magnetostriction^5^,^6^. It was shown that the proposed mechanism of magnetization control is robust even at elevated temperatures. All the clues point towards *magnetization dynamics* as a driving source for magnetization reversal asymmetry. It appears that both in the experiments^2^ and in simulations^5^ the timing is crucial. This led us to focus our investigation of the magnetization dynamics in order to uncover the underlying symmetry breaking mechanism in magnetization dynamics.

In this paper we explore the origins of the asymmetric behavior of magnetization reversal. The model system of our choice is the magnetic vortex - a well-studied magnetic structure that has potential for many applications. The magnetic vortex in soft magnetic nanoscale disks (nanodots) consists of a well known arrangement of magnetic dipoles that form a swirl in the plane of the disk with a small out-of-plane singularity in the center called a vortex core^7^. The sense of the dipole curl defines the vortex chirality and the direction of the dipole moment in the core defines vortex polarity. The range of geometric parameters for cylindrical nanodots having magnetic vortex configuration as equilibrium ground state in absence of applied magnetic field were determined theoretically^8^ as well as experimentally^9^. In case of Permalloy nanodots the lower limit is about 50 nm in diameter and about 40 nm in thickness. The genuine property of the magnetic vortex is its stability. It has very stable internal magnetization structure in many static and dynamic conditions. This results in successful description of the vortex via Thiele equation^10^. The vortex stability has inspired technological applications that span from using the vortex as an information carrier in non-volatile memories^11^,^12^ to active medium for medical applications^13^.

In certain cases the vortex stability could be detrimental for control of vortex state in a nanoscale system due to large energy budget needed to reverse either the chirality or the polarity of the vortex. Experimental results show that reduced symmetry might ease the control of chirality during vortex nucleation process^14^. The geometrical asymmetry induces energy splitting of the two chiralities in the presence of in-plane applied magnetic field. Meantime, the two polarities remain energetically degenerate. There has been several suggestions on how to control the polarity - from applying a static out-of-plane field^15^, to dynamically controlling the vortex nucleation by electromagnetic pulses^11^,^16^,^17^, microwaves^18^, or injecting current pulses^12^,^19^. We have shown previously that dynamic change (decrease) of the applied magnetic field also creates some bias for nucleation of a vortex with one preferred vortex polarity in Pacman-like (PL) structure^4^. In this paper we examine vortex nucleation in *static* external magnetic fields. In this case the vortex nucleation process is exclusively induced by temperature. Finite temperature induces fluctuations of the local effective fields that enable the system to overcome the energy barriers between local minima of the total energy functional. Taking into account Arrhenius theory only^20^, the probability of vortex nucleation is related solely to the system’s energy landscape and does not depend on dynamical effects. It is generally accepted that time evolution of magnetic structure in some cases does not follow Arrhenius law^21^,^22^. Deviation from Arrhenius law was observed in time and an additional parameter - “waiting time”, that was introduced into theory of thermal over-the-barrier crossing. In the section “Simulation of Pacman-like Nanodot” using numerical simulations of the vortex nucleation process we demonstrate that dynamics could induce “directional effects” that can not be observed by taking into account energetics only. These findings of the numerical simulations inspired our further search for possible physics responsible for such behavior. In section “Analytical Toy-model” we describe simple analytical one-spin model which has the same broken magnetization reversal symmetry behavior. In the section “Temperature Effects” we investigate the effect of temperature in our toy-model and we analyze whether the symmetry in the sense of probability of final state can be restored. We provide two sets of toy model parameters - the first set defines dynamics robust against thermal fluctuations that mimics the results presented in the section “Simulations of Pacman-like Nanodot”. The second model potential provides much weaker drift, so the elevated temperature can restore symmetric behavior of spin dynamics in probabilistic sense.

## Simulations of Pacman-like Nanodot

Vortex nucleation might be reached by various scenarios. In previous papers^3^,^4^ we analyzed magnetic structure of a magnetic dot with broken circular symmetry (Pac-man like nanodot) in slowly decreasing external magnetic field at zero temperature. The external field was decreasing until in-plane magnetic structure became unstable with respect to nucleation of out-of-plane local magnetization of the vortex state. We defined vortex nucleation field as the field when vortex core appears inside the PL-nanodot. This is a symmetry breaking process, because nucleated vortex core carries net out-of-plane magnetization called polarity. Polarity can have two possible orientations.

Presence of finite temperature makes the situation more complex. Local magnetization fluctuations induced by the temperature help overcome energy barrier of vortex nucleation at higher applied fields than in the case of zero temperature case. On the other hand higher temperature causes more vivid oscillations of the local moments, thus lowering mean value of saturation magnetization. This increase as well as decrease of the vortex nucleation field was indeed experimentally observed^23^. The temperature induced vortex nucleation has stochastic nature and thus the statistical description is proper choice. The first approximation for the rate of vortex nucleation can be made by using the Arrhenius law. However, it is known that there are some deviations from the theory in the case of magnetic systems^24^. This is because the Arrhenius law completely ignores the topology of the total energy surface (TES) in configuration space as well as any dynamical effects. The equation of motion for magnetic system is Landau-Lifshitz equation, a first order differential equation in time domain. Thus there is no “kinetic” energy, nor inertia. For this reason we use the term total energy surface (TES) instead of potential energy surface (PES) commonly used in Newtonian dynamics. It will be shown in the section “Analytical Toy-model”, that the TES topology is very important to properly describe vortex nucleation process.

**Figure 1:**
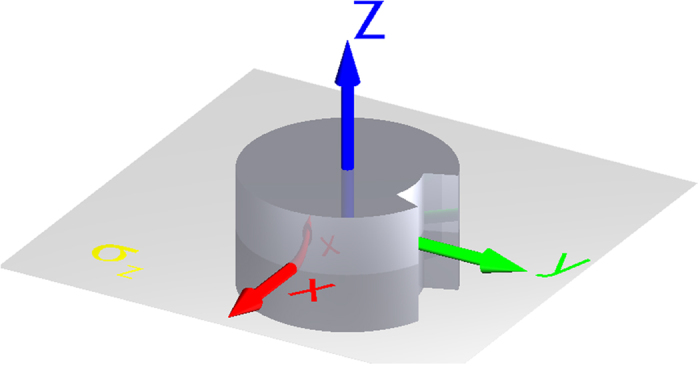
The geometry of the simulated Permalloy PL-nanodot. The outer diameter is 70 nm and the thickness is 40 nm. Missing sector has an opening angle of 45^°^. Important feature of the dot is its reduced symmetry (point group C_2v_) - the mirror plane *z* = 0 is noted as *σ*_*z*_. Applied magnetic field has only *x* and *y* components.

In this paper all the numerical simulations of the vortex core nucleation process were done using micromagnetic simulations^25^. The geometry of the PL nano-dot can be described as a disk with a missing sector. The outer diameter of nanodot is 70 nm and the thickness is 40 nm. The opening angle of cut-off sector is 45°, and the depth of the cut is one third of the radius (see the Fig. 1). The symmetry group of PL nanodot is C_2v_. The external magnetic field is applied only in *xy* plane. These facts guarantee equivalence of energies for magnetic structures with inverse *z* components (mirror symmetry *σ*_*z*_).

The vortex nucleation was obtained by following scenario: in-plane external magnetic field was slowly decreased to about 2 mT above the nucleation field at zero temperature. The magnetic structure was then relaxed to its metastable state without vortex by damped dynamics given by Landau-Lifshitz-Gilbert (LLG) equation. Then, the non-zero temperature was applied and the system was evolved until the magnetic vortex got nucleated. The time was measured starting from the moment when the non-zero temperature was turned on. Temperature effects were included into the LLG equation via Langevin-like stochastic term as described by Garcia-Palacios^26^ and implemented by Oliver Lemcke^27^. We stress that external field was fixed during the simulation, so the dynamic effect described in our previous study^4^ did not play any role.

**Figure 2:**
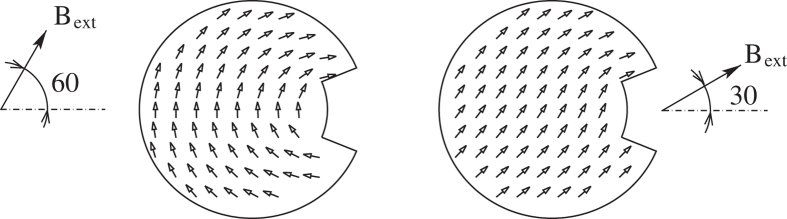
Magnetic structure of two initial states chosen as representative cases for *C*- (left) and *S*-states (right).

There are two qualitatively different kinds of magnetic states preceding the vortex state in slowly decreasing external magnetic field - *C*- and *S*-states - already identified earlier^4^,^28^. Interval of the angles of the external field with respect to the symmetry axis of PL nanodot depends on the geometry and it was subject of our previous study^28^. In this study only two particular angles were chosen as representative cases for the two kinds of initial magnetic configurations –60° for the *C*-state and 30° for the *S*-state (Fig. 2).

The initial magnetic structures are invariant to mirror operation *σ*_*z*_ - reflection with respect to the *xy* plane at *z* = 0 (see Fig. 1). Thus there is no net component of magnetization in *z* direction. The vortex state breaks this symmetry and results in non-zero net component of magnetization in *z* direction, thus setting up the polarity of the vortex. The vortex state has two—fold degeneracy corresponding to two possible vortex polarities. Assuming that both polarities have the same chance of nucleation, the probability of having *N*_−_ occurrence of negative polarity in the sampling of *N* realizations has the binomial distribution:


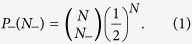


We performed a set of *N* = 100 simulations with different realizations of the random noise for each temperature and each initial condition. In Table 1 we show the results for the two initial states (*C*- and *S*-state) and various temperatures. Table 1 contains average vortex nucleation time *t*_*N*_, the sample variance of nucleation time 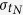, and the probability of realizing a negative polarity of the final nucleated vortex *P*_−_ = *N*_−_/*N*.

Based on the standard statistical analysis, the hypothesis that the vortex nucleate with equal probability for both polarities was accepted at the *α* = 0.05 level of significance for *C* initial state. The hypothesis was clearly incorrect in case of initial *S*-state. Vortex nucleation starting from the *S*-state nucleated always a vortex with negative polarity without any single exception. This leads us to believe that there is some deeper underlying mechanism for polarity choice in the equation of motion.

**Figure 3:**
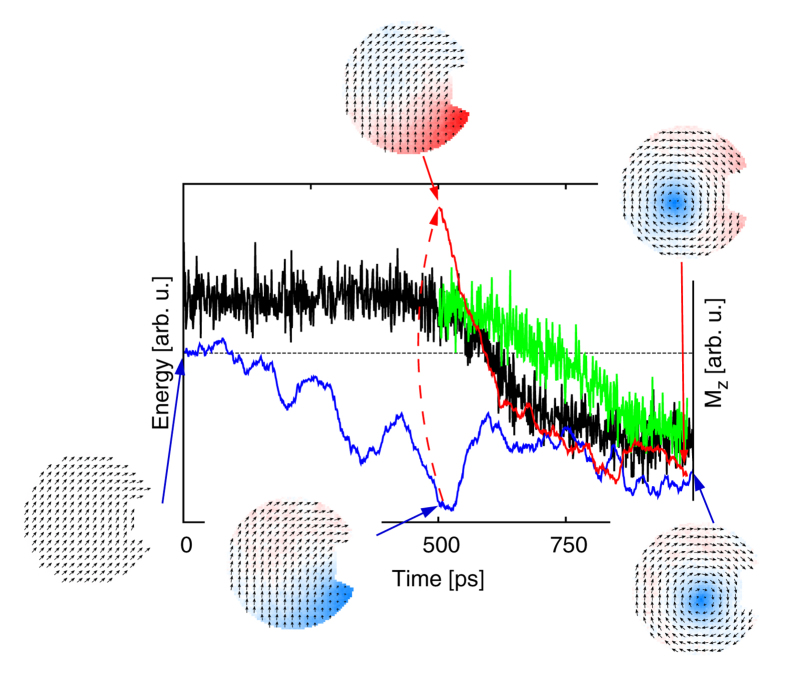
The time evolution of the vortex nucleation process at T = 300 K. At time *t* = 0 the system is in *S*-state (Fig. 2). The total energy is shown in black. The *z*-component of net magnetization is shown in blue. At time *t* = 500 ps the system is cloned and *z*-component of the magnetization is reversed. The total energy evolution of this replica is shown in green and the *z*-component of net magnetization in red. Time snap-shots of the magnetization field are also shown. The *z*-component of the magnetization is coded in color. The blue color indicates negative, red color positive *z*-component of **M** (see Figure 1 for coordinate system definition).

The intriguing observation was that net *z*-component of magnetization (defined as polarity) starts to evolve right after the total energy starts decreasing during the system evolution (at around *t* = 500 ps) (see our video in the Supplementary Material). At that moment, we interrupted the simulation and made a clone of the system with *z*-component of local magnetization reversed. The *C*-state case nucleated into vortex with inverse polarity, meanwhile the *S*-state dynamics led consistently into a state with negative polarity of the nucleated vortex (see the Fig. 3). This intriguing fact inspired us to construct a toy-model with similar properties and it is the focus of the next section.

## Analytical Toy-model

We consider a single spin **M** with fixed amplitude ||**M**|| = 1 and arbitrary orientation in 3D space. In this case **M** is single vector and not vector field. The total energy is then simple function rather than a functional. This makes model analysis much easier, since we avoid non-local interactions of dipole fields. We consider following particular form of the total energy function in dimensionless units:


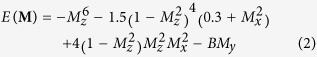



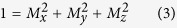


The form of the total energy function (2) could be representative of the energy of a single spin in specific material with crystalline anisotropy, or energy of macrospin of a nanodot that has a particular shape anisotropy and all spatial dimensions much smaller than exchange length (in order to fulfill condition (3)). The last term in the energy function is the term related to external field along *y* direction. The total energy function (2) is also invariant under transformation of symmetry group C_2v_. To fulfill constrain (3) automatically, it is useful to express TES in spherical coordinates:


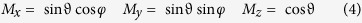



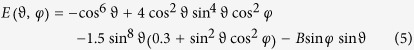


**Figure 4:**
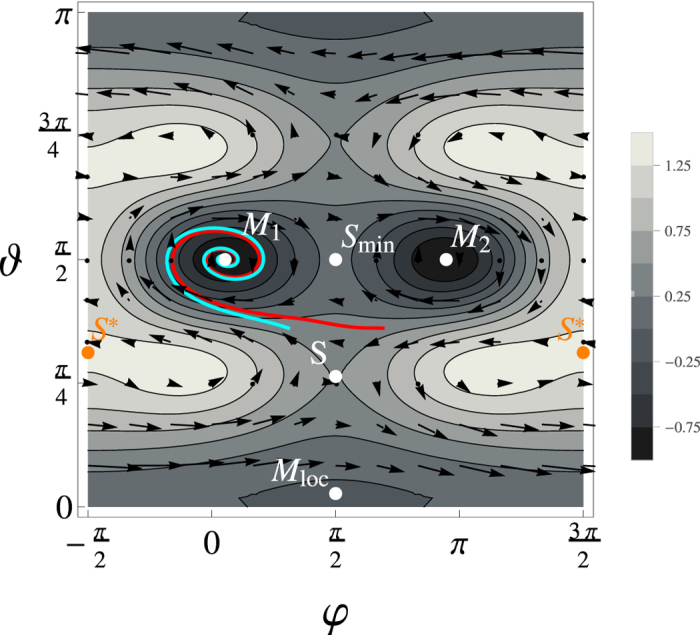
The map of the TES defined by Equation (5) for B = 0.5. The vector field generated by right-hand-side of equation (6) with *α* = 0.1 is shown by the arrows. Two zero-temperature trajectories are shown in blue and red lines. Position of some points is indicated in the graph: M_loc_ - local minimum in the vicinity of the pole, M_1_, M_2_ - global minima, S - saddle point (position of the lowest energy barrier) between local and global minima, S^*^ -other saddle point (higher in energy than S), S_min_ minimum energy barrier between global minima M_1_ and M_2_.

The map of TES with *B* = 0.5 is shown in Fig. 4. The role of the external field along *y* direction is to break symmetry of TES with respect to inversion *y* → −*y*. With moderate values of **B** the minimum energy path from local minima near M_loc_ has to pass through the saddle point *S* and not S^*^ (see Fig. 4 for notation). Strong enough fields (*B* > 1.706) eliminate the barrier between local and global minima. In the further analysis we will use value of *B* = 0.5.

The symmetry breaking mechanism is hidden in the dynamics. To demonstrate the symmetry breaking we consider trajectories starting from the vicinity of the saddle point *S*. The dimensionless Landau-Lifshitz equation describing our spin dynamics is expressed:


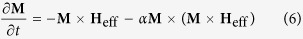



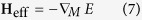


where *α* is the damping constant. Effective field **H**_eff_ could be obtained from the total energy function by taking a derivative of the total energy *E* with respect to magnetization **M**. It is important to note that the first term on the right hand side of equation (6) conserves the total energy because it changes magnetization perpendicular to the energy gradient. It means, that the first term induces dynamics along the curve of constant energy while the second term is dissipative and takes the direction of steepest decrease of the total energy.

Rewritten in spherical coordinates, it becomes a system of the two ordinary differential equations:


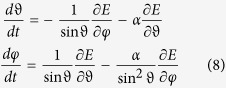


The explicit form of the right hand side can be obtained by embedding the expression for the total energy function (5) into definition of effective magnetic field (7). Qualitative understanding can be gained by looking at right-hand side of equations (8). The vector field defined by right-hand-side of equations (8) is shown in Fig. 4. Trajectories beyond the saddle point *S* are curling in clock-wise direction. If the dissipation is strong enough that after one turn the energy is not sufficient to overcome the energy crest at its lowest energy *S*_*min*_, the system evolves towards a minimum M_1_, without the possibility of reaching the minimum M_2_. To be more specific, the condition for no escape is defined by the energy of lowest laying saddle between global minima *M*_1_ and *M*_2_. Note that there are two possible trajectories of constant energy. The sign of the first term on the right hand side of LLG equation (6) is given by the sign of the gyromagnetic ratio and it would be positive for the case of proton spin dynamics resulting in the vector field nearby the global minima on picture (3) rotating counterclockwise.

## Temperature Effects

The last and the most difficult question is how the Fig. 4 is altered by thermal fluctuations. At the first sight the thermal fluctuations could be expressed as an addition of some random field to the right-hand-side of equation (6) (or to the vector field depicted on Fig. 4). Unfortunately, fluctuating field should appear as a cofactor of magnetization, which makes the analysis more difficult. Langevin-like version of Landau-Lifshitz equation takes the form^26^,^29^:


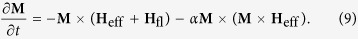


In the case of stochastic field with Gaussian uncorrelated distribution, the amplitude of the fluctuations, temperature and dissipation are related via the fluctuation-dissipation theorem^30^:


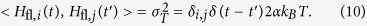


The *σ*_*T*_ is standard deviation of the normal distribution, *k*_*B*_ is the Boltzman constant, and *T* is the temperature. Expected property of the relation (10) is that fluctuations are enhanced at higher temperatures. The effect of fluctuations at large damping *α* is even stronger. The dissipation term proportional to *α* determines the angle between the tangent of constant energy line and the vector defined by right-hand-side of equation (8). If *α* is large, the trajectories passing in the vicinity of the saddle point at zero temperature follow the path of fastest energy descent. Thus in case of large *α* the system is more sensitive to fluctuations because the evolution of the system does not take place far enough from the bottom of the TES valley. Moreover, the damping constant *α* has the same role in the relation (10) as the temperature.

The TES given by equation (2) results in a strong drift in the vicinity of the saddle point and therefore does not exhibit the crossing from asymmetric to symmetric behavior in the probability distribution sense. This crossing from asymmetric to symmetric behavior was observed experimentally in magnetization measurements in nickel nanodots at high magnetic fields and low temperatures^1^. The problem in obtaining symmetric distribution function with TES given by equation (2) is following: if the simulation temperature is chosen to be too high, the trajectories passing in the vicinity of the minimum at **M**_2_ appear, but the temperature is high enough also for escape from minima **M**_1_. The system is then paramagnetic-like.

The above constraints led us to develop a TES with more flat energy landscape in the vicinity of the saddle point. Smaller gradient of the TES translates into smaller drift term **H**_eff_ with respect to the stochastic field **H**_fl_ according to the equations (7,9). Also, having deeper global minima decreases the probability of thermal escape. The new TES has the following form:


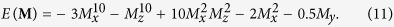


**Figure 5:**
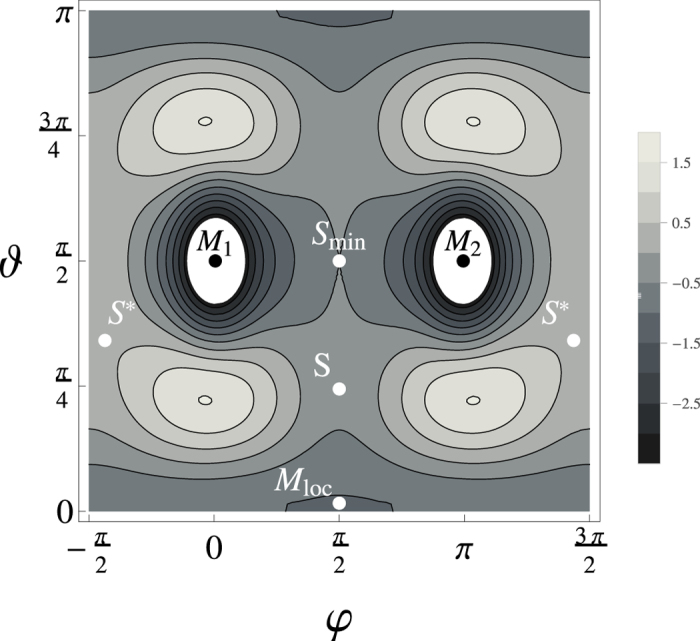
Map of the TES defined by Equation (11). Starting point of the simulations is local minimum labelled by M_loc_. Trajectories pass in vicinity of the saddle point S and reach one of the two global minima M_1_ or M_2_. Energy gradient in the region nearby the saddle points S and S_min_ is much smaller in comparison to the TES showed in Figure 4.

The last term, as in the previous case, could be interpreted as the external magnetic field in *y* direction. The purpose of this term is to break the symmetry of the saddle points S and S^*^. The other terms correspond to the symmetry of magnetic anisotropy. The energy profile in polar coordinates is shown in Fig. 5. For this potential we did simulations with various dispersions of the Gaussian noise *σ*_*T*_. The statistics is summarized in Table 2. The results show that at elevated temperatures the probability of reaching degenerate minima is again symmetrized. On the other hand, at low temperatures (*σ*_*T*_ < 0.25) the thermal fluctuations are not able to randomize the drift towards the minimum at M_1_.

## Conclusions

We have shown that particular shape of total energy functional in configuration space might lead to dynamics with broken symmetry. The topology of TES resulting in such broken symmetry dynamics has to have a single valley behind the lowest energy barrier (saddle point). The bottom of the valley should decrease monotonously. At some point single valley should split to two valleys leading to two different minima. The system trajectories starting from the saddle point are drifting away from the bottom of the valley due to precession term of LLG equation. The side of the valley is determined by sign of gyromagnetic ratio. When the single valley splits into two equivalent valleys, the system descends along the valley on its side. Thus the dynamics governed by LLG equation has an intrinsic symmetry breaking property.

Although the symmetry breaking by time evolution has been discussed in context of *time dependent* effective field^4^,^5^, here we have shown that even *static* total energy functional with realistic topology has the symmetry breaking property. Our work opens new directions in predictive design of magnetic nanoparticles based on particular shape of TES defined by the geometrical shape and specific magnetic material properties.

## Methods

The dynamics of magnetization field in magnetic nanodot involves numerical solution of the Landau-Lifshitz-Gilbert (LLG) equation together with solution of magnetostatic problem of finding magnetic induction in space with prescribed field sources. The approximation neglects the magnetic field generated by eddy currents.

The stochastic Landau-Lifshitz-Gilbert (LLG) equation in case of Pacman-like nanodot was solved with the use of micromagnetic simulation software^25^. The structure under investigation was a Permalloy Pacman-like nanodot (we used following material parameters: saturation magnetization **M**_*s*_ = 8.6 × 10^5^ A/m, exchange **A** = 1.3 × 10^−11^ J/m^3^, damping constant *α* = 0.5). Integration scheme used time step of 10^−14^ s. The Stratonovich interpretation rule for the time integration was used.

The toy model was described by dimensionless energy functions (2) and (11). The dynamics of the spin was described by Landau-Lifshitz equation (9). The time step used for time-integration was 10^−4^. The damping constant *α* = 0.1 was used in numerical calculations presented in Table 2.

## Additional Information

**How to cite this article**: Tóbik, J. *et al.* Asymmetry in Time Evolution of Magnetization in Magnetic Nanostructures. *Sci. Rep.* **5**, 12301; doi: 10.1038/srep12301 (2015).

## Supplementary Material

Supplementary video

Supplementary Legend

## Figures and Tables

**Table 1:** Summary of the outcomes of 100 simulation runs of vortex nucleation in PL nanodot from initial *C*– and *S*–states of magnetization.

**T[K]**	**C state**		**S state**	
	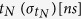	***P***_**−**_	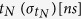	***P***_**−**_
300	3.11 (2.66)	0.56	1.14 (0.44)	1.00
600	2.13 (2.09)	0.49	0.97 (0.47)	1.00
900	1.68 (1.68)	0.53	0.99 (0.53)	1.00
1200	1.28 (1.17)	0.43	0.97 (0.57)	1.00

The average nucleation time *t*_*N*_, its sample variance 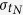 and the probability that the final state of nucleated vortex had negative polarity *P*_−_. The notation, *C*– and *S*– state reflects the initial magnetization arrangement as depicted in Figure 2.

**Table 2:** Summary of transitions from local minimum at *M*_*z*_ ≈ 1 to global minima at *M*_*x*_ ≈ ±1 of TES given by Equation (11).

*σ*_*T*_	**P(+)**	***t***_***N***_	*σ*_*tN*_
0.13	1.00	711.50	701.54
0.2	1.00	14.27	11.77
0.25	0.99	4.77	3.48
0.3	0.96	3.13	2.38
0.4	0.89	1.88	1.43

The damping constant *α* is 0.01. At different dispersions of the Gaussian noise *σ*_*T*_ the probability of reaching minimum with *M*_*x*_ ≈ 1 is P(+). The temperature *T* is related to dispersion *σ*_*T*_ via fluctuation-dissipation theorem (10). *t*_*N*_ is the average transition time to the final state and 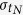 is the sample variance of the nucleation time. Sample size was *N* = 100 random realizations.

## References

[b1] Lok J. G. S. *et al.* Memory effects in individual submicrometer ferromagnets. Phys. Rev. B 58, 12201 (1998).

[b2] Ostler T. A. *et al.* Ultrafast heating as a sufficient stimulus for magnetization reversal in a ferrimagnet. Nature Commun. 3, 666 (2012). 22314362 10.1038/ncomms1666

[b3] Cambel V. & Karapetrov G. Control of vortex chirality and polarity in magnetic nanodots with broken rotational symmetry. Phys. Rev. B 84, 014424 (2011).

[b4] Tóbik J., Cambel V. & Karapetrov G. Dynamics of vortex nucleation in nanomagnets with broken symmetry. Phys. Rev. B 86, 134433 (2012).

[b5] Roy K., Bandyopadhyay S. & Atulasimha J. Binary switching in a ‘symmetric’ potential landscape. Sci. Rep. 3, 3038 (2013). 24154561 10.1038/srep03038 PMC3807113

[b6] Fashami M. S., Atulasimha J. & Bandyopadhyay S. Energy dissipation and error probability in fault-tolerant binary switching. Sci. Rep. 3, 3204 (2013). 24220310 10.1038/srep03204 PMC3826093

[b7] Shinjo T., Okuno T., Hassdorf R., Shigeto K. & Ono T. Magnetic vortex core observation in circular dots of permalloy. Science 289, 930 (2000). 10937991 10.1126/science.289.5481.930

[b8] Hoffmann H. & Steinbauer F. Single domain and vortex state in ferromagnetic circular nanodots. J. Appl. Phys. 92, 5463 (2002).

[b9] Chung S. H., McMichael R. D., Pierce D. T. & Unguris J. Phase diagram of magnetic nanodisks measured by scanning electron microscopy with polarization analysis. Phys. Rev. B 81, 024410 (2010).

[b10] Thiele A. A. Steady-state motion of magnetic domains. Phys. Rev. Lett. 30, 230 (1973).

[b11] Yu Y. S., Jung H., Lee K. S., Fischer P. & Kim S. K. Memory-bit selection and recording by rotating fields in vortex-core cross-point architecture. Appl. Phys. Lett. 98 052507 (2011).

[b12] Jenkins A. S. *et al.* Controlling the chirality and polarity of vortices in magnetic tunnel junctions. Appl. Phys. Lett. 105, 172403 (2014).

[b13] Kim D. H. *et al.* Biofunctionalized magnetic-vortex microdiscs for targeted cancer-cell destruction. Nat. Mater. 9, 165 (2009). 19946279 10.1038/nmat2591 PMC2810356

[b14] Schneider M., Hoffmann H. & Zweck J. Magnetic switching of single vortex permalloy elements. Appl. Phys. Lett. 79, 3113 (2001).

[b15] Jaafar M. *et al.* Control of the chirality and polarity of magnetic vortices in triangular nanodots. Phys. Rev. B 81, 054439 (2010).

[b16] Antos R. & Otani Y. Simulations of the dynamic switching of vortex chirality in magnetic nanodisks by a uniform field pulse. Phys. Rev. B 80, 140404(R) (2009).

[b17] Jain S. *et al.* From chaos to selective ordering of vortex cores in interacting mesomagnets. Nat. Comm. 3, 1330 (2012). 10.1038/ncomms2331 23271662

[b18] Lee K. S., Guslienko K. Y., Lee J. Y. & Kim S. K. Ultrafast vortex-core reversal dynamics in ferromagnetic nanodots. Phys. Rev. B 76, 174410 (2007).

[b19] Yamada K. *et al.* Electrical switching of the vortex core in a magnetic disk. Nature Materials 6, 270 (2007). 10.1038/nmat1867 17369832

[b20] see for example: Risken H. in The Fokker-Planck Equation, Method of Solutions and Applications. 2nd ed., (Springer-Verlag, Heidelberg, 1989).

[b21] Garanin D. A. & Chudnovsky E. M. Thermally activated resonant magnetization tunneling in molecular magnets: Mn12Ac and others. Phys. Rev. B 56, 11102 (1997).

[b22] González J. M., Ramrez R., Smirnov-Rueda R. & González J. Non-Arrhenius relaxation in micromagnetic models of systems with many degrees of freedom. Phys. Rev. B 52, 16034 (1995). 10.1103/physrevb.52.16034 9980985

[b23] Mihajlovic G. *et al.* Temperature dependent nucleation and annihilation of individual magnetic vortices. Appl. Phys. Lett. 96, 112501 (2010).

[b24] Suran G. *et al.* Evidences of non-Arrhenius magnetic relaxation in macroscopic systems: Experiments and related simulations Europhys. Lett. 41, 671 (1998).

[b25] Donahue M. J. & Porter D. G., *OOMMF User’s Guide, Version 1.0.* NISTIR 6376, National Institute of Standards and Technology, Gaithersburg, MD (Sept 1999).

[b26] Garca-Palacios J. L. & Lázaro F. J. Langevin-dynamics study of the dynamical properties of small magnetic particles. Phys. Rev. B 58, 14937 (1998).

[b27] Lemcke O. *Implementation of temperature in micromagnetic simulations*. Available at: http://www.nanoscience.de/group_r/stm-spstm/projects/temperature/download.shtml (Accessed: 1st December 2014).

[b28] Cambel V. *et al.* The influence of shape anisotropy on vortex nucleation in Pacman-like nanomagnets. J. Magn. Magn. Mater. 336, 29 (2013).

[b29] Brown W. F. Thermal fluctuations of a single-domain particle. Phys. Rev. 130, 1677 (1963).

[b30] Kubo R., Toda M. & Hashitsume N. in Statistical Physics 2. Nonequilibrium Statistical Mechanics. (2nd Edition, Springer-Verlag, Heidelberg 1998).

