# Supplementary material for: Asymmetry in Time Evolution of Magnetization in Magnetic Nanostructures
Source: Sci Rep. 2015 Jul 22;5:12301. doi: 10.1038/srep12301 (PMC4510517; doi:10.1038/srep12301)
Supplement: Supplementary Legend [file srep12301-s2.pdf]

Asymmetry in time evolution of magnetization in magnetic nanostructures

Jaroslav Tóbik* and Vladimír Cambel

Institute of Electrical Engineering, Slovak Academy of Sciences,

Dúbravská cesta 9, SK-841 04 Bratislava, Slovakia

Goran Karapetrov

Department of Physics, Drexel University,

3141 Chestnut Street, Philadelphia, Pennsylvania 19104, USA

Supplementary video

The supplementary video shows two types of magnetic vortex nucleation processes. "Symmetric" nucleation (nucleation from the C-initial state) is shown in the first part of video. Red (positive polarity) and blue (negative polarity) vortices nucleate with same probability. The second part of the video shows nucleation process starting from the S-state. This nucleation resulted always in the negative polarity of the vortex core. The robustness of the negative polarity is demonstrated by reverting the "z" component of the magnetization field at time (t=500ps), when energy barrier is already overcome and polarity already seems to be chosen. This is shown in the third part of the video. The statistics of the "numerical experiment" outcome is summarized in the table shown in the last part of the video.

*Electronic address: `jaroslav.tobik@savba.sk`
